# On Quackery

**Published:** 1802-04

**Authors:** 


### *To the Editors of the Medical and Phyſical Journal.*

GENTLEMEN,

A Great deal has been both written and ſaid upon the ſubject of Quackery, and I am afraid it is not in my power to ſay or write any thing to prevent it. Yet, it appears to me, that there is one thing which greatly tends to its being general:

general: Do we not frequently obferve in every country town, *This is the Patent Medicine Warehouse*; and, in fome druggift's Advertifements, *Physicians Prescriptions faithfully prepared.* But, let me afk, how are they prepared? Not by the mafter; but by a dirty boy, who is enough to ficken the ftomach of the pooreft, and who perhaps knows no more about Latin prefcriptions than a chimney-fweeper. The prefcription is made up at random, and the perfon difgufted with the idea of its being fo dirtily prepared. A paper is perhaps put into his hand, giving a detail of the wonderful cures performed by fome patent medicine; from the druggifts he runs to the Patent Medicine Warehoufe, which is perhaps at a bookfellers, where the article afked for is wrapped up clean; and not feeing the procefs of its making, he becomes a purchafer; and by fuch means, if the patient gets better, afcribes it to the quack medicines, and the article gets into repute.

I think another caufe of the increafe of quack medicines, is from the phyficians frequently ordering their *gratis* patients to take their prefcription to the druggifts, as they will there get it made up cheap; perhaps half the directions are written in Englifh, that the poor ignorant boy, or the mafter, who is fometimes a grocer and tea-dealer, &c. fhould underftand them.

I have fometimes been furprifed at feeing a number of prefcriptions lying at a druggift's fhop waiting to be made up, and which were for fuperior people; and upon minute inquiry, have received an anfwer fuch as this: Dr. —— faid I might take it to the druggift, it would be made up as cheap as at Mr. ——'s the apothecary. The regular bred apothecary is thus left out, who has a good education, and attended to chemiftry, &c. whilft the druggift's boy is to make up medicines of which he does not know the ufe of one ingredient, and fometimes, by miftakes, has occafioned the death of the patient. I am likewife forry to fee Quackery fo much reforted to by the great, who fly to Quack Doctors who cure the gout by hot bathing; we have hot bath doctors and cold bath doctors, all for one complaint!

Through the medium of your highly refpectable Journal, I could wifh to caution all phyficians, furgeons, &c. from fending their prefcriptions to druggifts, but let them go to the regular apothecary; the medicine would be faithfully prepared; and it would be of fervice to the patient, and be one means of leffening the increafed tendency of the common people to run after Quacks.

I do not doubt but you, Gentlemen, will think with me, in this age of Quackery, every caution ought to be ufed to prevent vent its increaſe; "That it has increaſed, is increaſing, and ought to be diminiſhed."

If you think the above worthy a place in your Journal, at ſome future period will give you a longer Eſſay on the Dangers of Quackery. I am, &c.

*Leeds, Feb.* 27, 1802. ANTI-QUACKERY.

